# Psychological autopsies: religious and spiritual factors for suicide in cases and controls

**DOI:** 10.3389/fpsyt.2024.1419669

**Published:** 2024-10-16

**Authors:** Syeda Ayat-e-Zainab Ali, Tamkeen Saleem

**Affiliations:** ^1^ Department of Psychology, International Islamic University, Islamabad, Pakistan; ^2^ Department of Clinical Psychology, Shifa Tameer-e-Millat University, Islamabad, Pakistan

**Keywords:** suicide, psychological autopsy, religious, spiritual, Muslims, Pakistan

## Abstract

**Introduction:**

In recent years, there has been a growing body of evidence supporting the notion that spirituality and religion can improve one’s physical and mental health. Studies show that religion and spirituality play a significant role in the suicidal path. Considering the dearth of data on the patterns of suicidal thoughts, acts and related factors in Pakistan, a country with a large Muslim population and stigma with sociocultural perceptions of religious notions, the current study investigated the role of religious and spiritual factors of suicide using psychological autopsies.

**Method:**

Purposive sampling was used to gather retrospective data records from police stations and hospital forensic departments in Rawalpindi and Islamabad, Pakistan, between January 2021 and July 2022. In total, 60 samples (30 cases and 30 controls) were used in this autopsies investigation. As with the majority of case control studies, the study sample cases were matched with the controls. At least two close bereaved family members served as the primary information sources, and data was gathered using the Semi Structured Interview Protocol for Psychological Autopsies.

**Results:**

According to statistical analysis, both the suicide cases and the controls were comprised mostly of Muslim individuals. They follow Islam and hold the view that suicide is prohibited, forbidden, and haram in Islam. Regarding spirituality and religion, the majority of cases, according to the respondents interviewed, were not spiritual at all, while the controls are slightly spiritual. While the majority of both cases and controls had reduced interest in or practice of religious activities over the past year, a higher number of cases rarely performed and attended the religious services/activities as compared to controls.

**Discussion:**

It was thus evident that the cases lacked significant engagement in spiritual and religious activities, and their attendance at such gatherings had notably decreased in the year preceding the suicide attempt. This decline in involvement in spiritual and religious practices might be linked to decreased levels of satisfaction, sense of belongingness, which could raise the risk of suicide. Therefore, in nations where Muslims predominate, culturally relevant suicide prevention initiatives including spiritual and religious treatments aimed at reducing the risk of suicide should be considered.

## Introduction

Research and services related to mental health, among other fields, have largely shown an increase in interest in subject of spirituality and religiosity (1–4). These terms are bracketed and differentiated in Western academia such that spirituality is understood to be a broader concept that includes the individual search for understanding answers to fundamental questions about life, meaning, and relationship with the sacred or transcendent, and religion is seen to consist of beliefs, practices, and rituals related to the transcendent (5, 6). Studies have indicated that spirituality and religion are typically linked to improved mental health (7). Increased levels of spirituality or religion have been linked to decreased levels of depressive symptoms (8), eating disorder symptoms (9, 10), negative symptoms of schizophrenia (11), perceived stress (12), risk of suicide, and personality disorder (13). Patients’ general quality of life is generally improved by religion and spirituality (10, 11). It has been demonstrated that spirituality or religion serves as a protective factor and improves adherence to psychiatric treatment (14). Additionally, better psychological health is linked to a greater degree of conviction in one’s belief system (10, 15).

The epidemiological profile of suicide also seems to be influenced by spiritual and religious variables (1, 2), particularly in nations where suicide is prohibited by religion. Suicide is a categorically forbidden act in Islam (16). While all monotheistic religions forbid suicide, Muslims frequently condemn suicide even more harshly, calling it an unforgivable sin. Muslims often believe that persons who die by suicide are not allowed to enter Heaven, even though the Qur’an makes no mention of this (17). According to Alothman and Fogarty (18), in Muslim-predominant nations, the median male-to-female suicide rate ratio was 2.5 in 2015—the second-highest ratio behind Christian-predominant countries, at 3.3. However, whether the percentage of Muslims and the male-to-female suicide rate ratio are correlated remains uncertain. According to a recent study, Muslims are the least permissive of all major religions regarding suicide, regardless of religiosity, and there is a correlation between permissiveness and national suicide rates (19).

The following of the Islamic way was the reason for liberation of the land known as Pakistan and therefore Islamic values and instructions form the foundation of Pakistani legislation and Muslims make up the majority of the population in Pakistan. Suicides and attempted suicides were punishable by state law up until December 2022, Section 325 of the Pakistani Penal Code, 1860 (PPC), which carried a maximum sentence of one year in prison and/or a significant fine. Furthermore, the law stipulates situations involving suicide attempts may only be handled at specific medicolegal centers (MLCs) or required registration at an MLC if treated at a private hospital. Nevertheless, neither of these guidelines was adhered to rigorously in practice. Blackmail and extortion of suicide attempters and their families by corrupt officials was frequent, despite the fact that the heavier penalties permitted under PPC Section 325 were rarely applied (16, 20).

A shift in the legal landscape has started when suicide attempts are no longer considered crimes as of December 2022. But since implementation is still a ways off, a lot of the obstacles posed by the previous law likely still stand. In Pakistan, treating suicide thoughts and actions is hampered not just by medical and legal restrictions but also by societal and religious ones, such as pervasive stigma (20). The age-standardized suicide death rate in Pakistan for 2019 was projected to be 9.8 per 100,000 people in the most recent World Health Organization (21) report, however this is probably an underestimate (21). Pakistan has conducted some study on the epidemiology of suicidal thoughts and behaviors, but the nation lacks an official, standardized monitoring system to gather these data on a national scale (16). The stigma directly impacts such an undertaking and data gathered is not reflective of reality.

According to earlier research, a significant percentage of medical admissions for suicide attempts in Pakistan are made by young people under 30 (22). Prior research on suicide in Pakistan has revealed risk factors for self-harm, including being a woman (particularly if married), having a mental illness, and being unemployed (23). Research has indicated that suicidal ideation are common among Pakistani college students (24) and teenagers who attend school (25). Furthermore, the most prevalent mental health issues were detrimental substance use and major depressive illness. In addition, self-harming thoughts, irritability and aggression, low self-esteem, treatment non-adherence, familial conflicts, and financial hardship have all been linked to young adults who have attempted suicide (26). In Pakistan’s north, suicide rates are startlingly rising, especially in the Valley of Chitral (27). Rahnuma et al. (27) asserted that Northern Pakistani culture and society underwent a significant transition due to fast social change, moving from pastoral to agricultural life, then to government and corporate employment, and finally towards a merchant society that was succeeded by a capitalist society (28). As a result of this change, individualism replaced collectivism as the prevailing value in religious society. People are getting less social help and are left to deal with their issues on their own. The primary risk factors for suicide, alienation and dissatisfaction, are frequently fueled by this isolation (29).

While there is evidence that religion and spiritual beliefs can prevent suicide (30), the empirical evidence varies. While some studies find that religion and spiritual beliefs can prevent suicide (2, 31, 32), others find that they are risk factors (33), and still others find no correlation between religion and suicide risk (2, 34). For example, Akotia et al. (35) found that spiritual difficulties were encountered by attempted survivors either before or during their hospitalization and while receiving medical care in Ghana. While going to church might help prevent suicide, there is evidence that poor mental health and ultimately suicide are linked to interpersonal issues with churchgoers, doubts, and fear of God’s wrath (5, 36).

Suicide and religion are both complex phenomena, which makes their relationship difficult to understand. Just as suicide has multiple dimensions (ideation, attempt, completion), religion also has multiple aspects (affiliation, participation, doctrine) (2). Spirituality and religion have a psychological impact on health. Cognitive, emotional, self-and-identity, and socio-psychological phenomena, are among these psychological factors (37, 38). Pargament (39), for example, provides a list of religious coping mechanisms, like internal locus of control, prayer, meditation, and self-directing style, that can be used to deal with stressful life events. Better physical and mental health outcomes are found to be associated with other psychological characteristics like self-esteem, self-efficacy, and strength of will, which have been linked to religious commitment in some research (38). In addition to that, the formation of a person’s spiritual identity and the integration of spirituality into a sense of self are significant aspects of spirituality that are also associated with wellness (40). Therefore, spirituality can be understood as a journey that offers a person a variety of objectives, including self-improvement, a sense of connection to God or the sacred, and the cultivation of certain values or the performance of particular rituals as a means of achieving more significant objectives (38). Pargament (39), Hood et al. (41), have all described religion as a symbolic cosmos. A person’s belief system is especially crucial during a crisis, like when they are contemplating suicide, since it can offer support in comprehending the challenging circumstances and accepting the sad and stressful events that have occurred, potentially overcoming pressures (42). Thus, understanding the presence of existential components (such as spirituality, religion, meaning, etc.) in suicidal behaviors as risk factors or resources for recovery is crucial for understanding suicide and its prevention in context (43, 44). Suicide frequently happens when someone decides that living is no longer desirable as a result of excruciating pain and a lack of coping mechanisms. People value religion and spirituality globally. They establish belief systems and offer prosocial, socially adaptable guiding principles (45). Further emotional and spiritual states and attributes that Islam supports are patience, gratitude, never losing hope, dependence on God, contentment, and peace. Many research (46–48) suggest that each of these stages provides the individual with attitudes, emotions, and feelings that could potentially assist prevent suicide (1).

According to Stratta et al. (49), negative spiritual coping also raises the risk of suicide. More research on the connection between spirituality and religion and suicidality is necessary in light of these apparently contradictory results. Currently, the most direct method to examine the relationship between specific factors and suicide is the psychological autopsy method, which is based on interviews with at least two informants close to the deceased and reviewing the data records, either medical or police report (50). Therefore, the study’s goal was to look into the role that religion and spirituality had in suicide cases and controls using psychological autopsy to explore the influence of these factors comprehensively.

### Aim

Of all suicide deaths worldwide, an estimated 60% is accounted by suicide in Asian nations, which is a substantial share of these deaths (51). Asia, with one-third coming from South Asia, and the African area are home to the bulk of Muslim countries. Low- and middle-income countries (LMICs) comprise the majority of them (17). Suicides happen everywhere and in all cultures, although a disproportionate amount happen in low- and middle-income countries (LMICs), accounting for more than 88% of all suicides (21, 52).

With a population of more than 213,000,000, the Islamic Republic of Pakistan is an LMIC on the South Asian subcontinent and the fifth most populated nation globally (53). In Pakistan, religion is a commonly held belief. There are multiple languages, civilizations, subcultures, ethnic groups, and religious sects in the nation, which is divided into four provinces (Punjab, Sindh, Balochistan, and Khyber Pakhtunkhawa) (22). Since gaining independence, the nation has had to contend with significant issues including an unstable political system, inadequate governance, and persistently low social and health indicators (22, 54). Despite the fact that there are only 0.19 psychiatrists per 100,000 people in Pakistan, mental problems account for more than 4% of all diseases, according to the World Health Organization, and 24 million people are thought to require mental healthcare services (20). Ninety-six percent of Pakistanis identify as Muslims, and Islamic religious traditions are prevalent in both the public and private sectors of daily life (20). Numerous economic, societal, and religious issues have been found to contribute to Pakistan’s poor mental health, which is further hampered by the country’s small labor force (54). In 2018, 39.8% of people were living below the lower middle-class poverty line. The actual figure might be greater (20).

According to the World Health Organization (21), there were 8.9 suicides per 100,000 people in Pakistan alone in 2019 (13.3% of males and 4.3% of females). This means that one person dies by suicide per hour, or between 15 and 35 individuals each day (55). According to a study examining crude suicide rates, suicide rates in Peshawar ranged from 0.43 per 100,000 people (1991–2000) to 2.86 per 100,000 people (2006) in Rawalpindi. With a gender ratio of 16:1, Rawalpindi, Punjab, had the highest rates of crude suicide. Compared to women, men are more prone to complete suicide (23). According to a recent study, Punjab accounted for the majority of completed suicides (79.7%) (22). Hence, the present study aimed to gather data from Rawalpindi, Islamabad, and its catchment areas. Apart from data on prevalence and risk factors, nothing is known about the role that spiritual and religious factors play in suicide. As of now, no single study in Pakistan has conducted matched case control psychological autopsy to examine the role that spiritual and religious elements played in suicide. It is imperative to comprehend the impact of spiritual and religious elements contributing to suicide, given the widespread occurrence of spiritual and religious beliefs worldwide, the high suicide rate, and the paucity of research on suicide in Pakistan. Taken together, this psychological autopsy study sought to shed light on the role that spiritual and religious factors played in suicide among cases and controls, given the mounting body of research indicating both a positive and negative relationship between religion and suicide as well as the pressing need to put suicide prevention measures into place.

## Method

### Sample and study design

This study followed a retrospective case control study design (56) to collect data on cases and controls in order to perform a psychological autopsies of suicide. Based on data records, the Police Stations and hospital forensic departments in Rawalpindi and Islamabad, Pakistan, provided a list of occurrences that were determined to be suicides following a medico-legal autopsy. Additionally, information about the control group which comprised of suicide attempt survivors from January 2021 to July 2022 was also obtained from the police stations and hospital forensic departments. Purposive sampling was therefore the method used to select the cases and controls. A sample of 24 cases and 24 controls was computed by maintaining the odd ratio (RR, also known as the risk ratio, or OR) at 7.5, significance at 0.10, with a 95% confidence interval and 80% power (57). Thus, the current study contained a sample size of 60 (n=30 cases, n=30 controls). The study included controls and patients with ages ranging from 10 to 35. As with the majority of case control studies, the study sample was matched in terms of age (± 2 years), gender, year of death (± 2 years), and length of hospital stay to controls who had died by suicide. At least two close bereaved family members (parent, sibling, or partner) who were at least 18 years old served as the primary information source for cases. The duration of the study was 2021-2023.

### Psychological autopsies method

Research on psychological autopsies makes use of the advantages of qualitative and quantitative techniques to produce a comprehensive picture of the life and circumstances surrounding the death of the person. Hence, research on psychological autopsies usually employs a mixed-method approach, integrating qualitative and quantitative components to comprehensively examine and comprehend the factors that led to an individual’s demise (58). Similar to biological autopsies, a psychological autopsy uses a checklist to understand the mental state of the deceased shortly before they pass away. A portion of this information is biographical (birthdate, occupation, marital status, relationship status, and any secondary data like a criminal record or family history), personal (such as any history of drug or alcohol abuse, known stresses, lifestyle, relationships, etc.), and information obtained through interviews with the deceased’s family members (59, 60). Interview and document data are frequently categorized into categories so that they can be quantified. Researchers may, for instance, calculate the prevalence of particular risk variables, such as past attempts at suicide, mental health conditions, or drug abuse. By statistical analysis, it is possible to find trends, differences, correlations, or risk factors related to suicide in quantitative data obtained from medical records, demographic data, and other structured data sources. While the quantitative data gives the qualitative observations more rigor and generalizability, the qualitative insights give the quantitative findings depth and context. Researchers can get a more thorough and nuanced knowledge of the factors that led to the death by combining the two approaches.

### Instrument

The American Association of Suicidology for Psychological Autopsy developed and updated a semi-structured interview technique, which was comparable to the one used in earlier psychological autopsy research (61, 62). The procedure included the gathering of deceased’s or control’s demographic data as well as additional details about their level of spirituality and participation in religious activities.

### Interview techniques

The family of the person who had died by suicide and controls was first contacted via a cell phone call. Following the initial communication, a culturally appropriate discussion of the study’s aims and procedures took place, and a time for the semi-structured interview was scheduled. Of the interviews, 27 (45%) took place in front of the police, while 33 (55%) take place in the consultant’s duty room at the hospital’s emergency room. The parents, spouses, and siblings were the primary informants. For each individual case, two family members were also present. The first author conducted all of the interviews. The interview was conducted between six and twelve months following the suicide. Typically, interviews lasted between forty-five to fifty minutes.

### Statistical analysis

Data gathered from interviews, and documents are further categorized into categories so that they can be quantified. For this purpose, we used IBM SPSS version 25 for the statistical analysis. P < 0.05 was used as the statistical significance threshold. Frequency and percentages were used to display the demographic characteristics. For categorical variable cross tabulations, Pearson chi-square was employed.

### Ethics statement

The research was carried out in conformity with the Helsinki Declaration of 1975, which was updated in 2008. The International Islamic University’s (permission number 26/03/2021/42DPEC) ethics committee for the psychology department in Islamabad, Pakistan, gave it their approval. Every informant provided written consent after being informed, and the study was registered (NCT05097690).

## Results

After matching the cases (n = 95) and controls (n = 96), 191 of the 334 records that were initially found at the police stations and hospitals in Rawalpindi and Islamabad, Pakistan, were contacted; 63 of them took part in the study. Moreover, 3 out of 63 were excluded from the analysis because of doubts about the information that was provided.

**Table 1** presents a sociodemographic profile of suicide cases and controls. Among the 60 cases and controls, a higher percentage of suicide cases and controls (n = 12 (40.0% cases, n = 10 (33.3% controls)) are observed in the 30-34 age group as compared to other age groups. 33.3% (n=10) of the controls and the majority of suicide cases are Punjabi as per the ethno-linguistic group. Males (n = 20, 66.7%) make up the majority of cases, while females (n = 20, 66.7%) make up the controls. Higher percentages of cases (n=19, 63.3%) and controls (n=22, 73.3%) are associated with lower socioeconomic level. The majority of cases (n=15, 50.0%) are single or never married, while a larger percentage of controls (n=15, 50.0%) are married. A significant percentage of cases (n = 18, 60.0%) are part of the nuclear family system, whereas controls (n = 17, 56.7%) are part of the joint family system and live in an urban region (cases = 22, 73.3%, Controls = 20, 66.7%).

**Table 1:** Socio demographic profile of cases and controls.

Variable	Category	*Cases*		Controls	
		*f *	%	*f *	%
Age	10-14 15-19 20-24 25-29 30-34	1 5 7 5 12	3.3% 16.7% 23.3% 16.7% 40.0%	0 7 4 9 10	0.0% 23.3% 13.3% 30.0% 33.3%
Gender	Male Female	20 10	66.7% 33.3%	10 20	33.3% 66.7%
Ethnolinguistic groups	Punjabi Urdu Speaking Pashtoon Sindhi Balochi Hindko Kashmiri Gilgiti Potohari Saraiki	10 6 4 1 1 3 0 2 0 3	33.3% 20.0% 13.3% 3.3% 3.3% 10.0% 0.0% 6.7% 0.0% 10.0%	10 1 6 1 2 5 1 1 1 2	33.3% 3.3% 20.0% 3.3% 6.7% 16.7% 3.3% 3.3% 3.3% 6.7%
Socio Economic Status	Lower Middle Upper	19 11 0	63.3% 36.7% 0.0%	5 22 3	16.7% 73.3% 10.0%
Marital Status	Single, Never Married Engaged Married Divorced Widowed	15 2 12 0 1	50.0% 6.7% 40.0% 0.0% 3.3%	11 3 15 1 0	36.7% 10.0% 50.0% 3.3% 0.0%
Family Type	Nuclear Joint	18 12	60.0% 40.0%	13 17	43.3% 56.7%
Living Area	Rural Urban	8 22	26.7% 73.3%	10 20	33.3% 66.7%

To determine whether there is a statistically significant difference between the cases and controls of suicide in the areas of spirituality and religiosity, chi square statistics were used. Given that most of the cases and controls are Muslims and practice Islam, **Table 2**’s results indicate that there is non-significant difference between them in terms of identification with a particular religion and religious beliefs regarding suicide death (X2(1, n=60) = 1.017, p = n.s.). Regarding spirituality and religiosity, the majority of cases exhibit no spirituality at all/or low levels of spirituality, while the controls exhibit a slight levels of spirituality (X2(2, n=60) = 13.644, ***p<001). A larger percentage of cases and controls participated in religious activities and attended services rarely, X2(5, n=60) =15.160, **p<.01, while the majority of cases and controls reported a reduction in their degree of participation in religious activities over the previous year, X2(1, n=60) =6.239, *p<.02.

**Table 2:** Religious and spiritual factors for suicide in cases and controls (N=60).

Factors	Categories	Cases		Controls		*x* ^2^	*P*
		*f *	%	*f *	%		
Religion	Islam Christian	29 1	96.7% 3.3%	30 0	100.0% 0.0%	1.017	.500
Levels of Spirituality	Moderately Spiritual Low Spirituality Somehow/Slightly Spiritual	1 25 4	3.3% 85.3% 11.4%	3 11 16	10.0% 36.7% 53.3%	13.644	.001***
Engagement in Religious Practices/Activities	Daily Once a Week Once or twice a month A few times a Year Rarely Once a Year or Less	0 0 1 7 21 1	0.0% 0.0% 3.3% 23.3% 70.0% 3.3%	1 4 7 2 13 3	3.3% 13.3% 23.3% 6.7% 43.3% 10.0%	15.160	.01**
Changed level of participation in Religious practices over the Past Year	Decreased Remained the same	25 5	83.3% 16.7%	16 14	53.3% 46.7%	6.239	.02*
Religious beliefs in family regarding Suicide	Prohibited Tragic Act	29 1	96.7% 3.3%	30 0	100% 0.0%	1.017	.500

χ^2^, Chi Square Statistics, Fishers test statistics (*p<.05,.500), **p<.01, ***p<.001.

## Discussion

The relationship between religion and spirituality and suicide has mostly been investigated in terms of its capacity to prevent suicide as well as moderating risk factors for suicide, like depression (63). The current research has examined the impact of spiritual and religious elements on suicide, in an effort to add to the body of existing literature and fill in knowledge gaps with particular regard to the Muslim population. In the current study, cases were found to have low levels of spirituality, while controls have been found to be somewhat spiritual. This is significant difference considering cases completed suicides and controls did not. The results align with a recent study wherein, after adjusting for sociodemographic variables, low level of spirituality was found to be a significant independent factor for an increased risk of suicide attempts (64). Spirituality has been linked in a number of studies to improved mental health and a decreased risk of suicide (65, 66). Low spirituality, on the one hand, makes it more difficult for people to use healthy coping mechanisms when under stress (67). However, other researchers contend that spiritual practices support “resiliency” (68). Furthermore, it’s critical to remember that spirituality is a very unique and individualized component of the human experience, and that it can have a wide range of effects on mental health which may be hard to objectively study particularly in retrospect from second-hand sources such as relatives who are informants. Nevertheless, some people find that spirituality gives their lives meaning and purpose (69). Strong senses of purpose have been linked to lower suicide rates because they may serve as a deterrent to hopelessness (70).

Being a part of spiritual or religious societies frequently offers a social support system. This feeling of belonging and community can improve mental health and lower the likelihood of suicidal thoughts (69). It was found in the current study that neither the patients nor the controls significantly participated in spiritual or religious activities, and that their attendance at these events had significantly declined in the year before the suicide attempt. Lower satisfaction levels could be linked to this decline in participation, which could increase the risk of suicide conduct (43). According to earlier research, people who identify as religious (71, 72), attend religious services more regularly (73, 74), and believe that religion plays a significant role in their lives (75) have lower rates of suicide attempt.

The degree to which a particular religion condones suicide may have a mediating effect on the potential protective effects of religion against suicide, both on an individual and societal level (76). This aligns with ecological research, which shows that suicide rates are lower in nations where the state actively promotes religious beliefs and higher in those where they are (77, 78). Additionally, it aligns with research conducted at the individual level that have discovered factors such a lack of religious belief to be a suicide risk factor (79). Therefore, there is ongoing discussion regarding the precise processes via which religion can prevent suicide (2).

Furthermore, among the major world religions, Muslims were shown to have the lowest level of permissiveness toward suicide, irrespective of their level of religiosity (19). National suicide rates were also found to be correlated with permissiveness (78). Despite their belief that suicide is forbidden or haram in Islam, both the cases and the controls in this study attempted suicide. It might be because of its links to elements of individual or social order, such wellbeing (80) or social order, including a lack of social support (81), as well as varying coping mechanisms (39). On the other side, it has been determined that factors connected to religion can affect views regarding suicide, such as shame, being left out of groups, or spiritual discontent (82). Additionally, Muslims who place no emphasis on God and pray infrequently have a more positive outlook on suicide, whereas Muslims who place a high value on God have the lowest suicide rates (19).

As religion may provide believers with a worldview about life and themselves, deeply spiritual and religious people tend to be more upbeat and confident in their ability to overcome challenges (83, 84), which makes them less likely to act suicidally (83, 85). It would be a useful avenue to investigate the connection between spirituality and religion and suicidal intent and ideation (86) in suicide survivors in future studies. In the future, additional specialized statistical research might be carried out to get a more accurate understanding of differences across particular religions (e.g., compare the Hindu, Muslim, and Christian communities living in Pakistan). Because of the limited sample size, caution should be used when extrapolating the study’s findings.

The results recommend including religious and spiritual components in general mental health care as well as programs that prevent and address suicidal conduct. Future studies are still needed to fully understand this issue from a qualitative standpoint. This will enable us to examine the discourse surrounding the irreversible and early disruption of life that is suicide and gain a deeper understanding of the religious and spiritual narratives of individuals who practice Islam and other religions. Due to the ease of accessibility, the data was gathered from Rawalpindi, Islamabad, and its catchment areas, hence future studies should conduct Psychological autopsies in other places of Pakistan like Gilgit Baltistan and Chitral. Also, in order to provide information on the unique religious and cultural context of suicide in Pakistan, it is also critical to improve earlier theoretical frameworks about the contribution of causes to suicide in the setting of Pakistan. Additionally, while screening individuals who may be at risk of suicide, an evaluation of their level of spirituality and religion in addition to mental risk factors may be advised. In psychiatric clinics, it is advised that all patients be asked to provide a brief history of their religious and spiritual practices.

## Conclusion

In Asia, suicide is a serious and intricate problem. The typical profile of suicide described in the scientific literature is different from the epidemiological profile found in Asian countries, as the former is typically based on research undertaken in European and American countries. The intricate web of socioeconomic, cultural, and religious elements in Asian countries may help to explain this, at least in part. The study’s findings are consistent with the idea that spiritual and religious influences have a significant impact on suicide thoughts and actions. According to available data, suicide is influenced by low spirituality, a decline in religious membership and attachment, and a drop in engagement in religious activities. Suicide prevention efforts in Muslim nations obviously need to take these contextual difficulties into account, as awareness of these elements can be very helpful in policies and procedures aimed at preventing suicide.

## Data Availability

The original contributions presented in the study are included in the article/supplementary material. Further inquiries can be directed to the corresponding author.
